# Combined genicular artery embolization and genicular nerve block to treat chronic pain following total knee arthroplasty

**DOI:** 10.1186/s42155-023-00409-3

**Published:** 2024-01-03

**Authors:** Wenhui Zhou, Eric Bultman, Lisa A. Mandl, Nicholas J. Giori, Sirish A. Kishore

**Affiliations:** 1 Department of Radiology, Stanford University Medical Center, Stanford, CA USA; 2 Division of Rheumatology, Hospital for Special Surgery, New York, NY USA; 3 Department of Orthopedic Surgery, Stanford University Medical Center, Stanford, CA USA; 4 Orthopedic Surgery Service, VA Palo Alto Health Care System, Palo Alto, CA USA; 5 Interventional Radiology Service, VA Palo Alto Health Care System, Palo Alto, CA USA

**Keywords:** Genicular artery embolization, Genicular nerve block, Knee arthroplasty, Chronic TKA pain

## Abstract

**Background:**

Chronic knee pain after total knee arthroplasty (TKA) is a common complication that is difficult to treat. This report aims to highlight the benefit of combining embolotherapy and neurolysis intervention for symptomatic relief of post-TKA pain in a patient with long-standing pain refractory to conservative management.

**Case presentation:**

A 77-year-old man who had previously undergone left knee arthroplasty has been grappling with worsening knee effusion and debilitating pain, resulting in limited mobility and progressive musculature deconditioning over a 20-year period. Diagnostic arteriography showed marked diffuse periarticular hyperemia around the medial and lateral joint spaces of the left knee, along with capsular distention. The patient initially underwent microsphere embolization to selectively target multiple branches of the genicular arteries, achieving a 50% reduction in pain at the one-month follow-up. Subsequently, the patient underwent image-guided genicular nerve neurolysis, targeting multiple branches of the genicular nerves, which led to further pain reduction (80% compared to the initial presentation or 60% compared to post-embolization) at the one-month follow-up. This improvement facilitated weight-bearing and enabled participation in physical therapy, with sustained pain relief over the 10-month follow-up period.

**Conclusion:**

The combination of genicular artery embolization and genicular nerve block may be a technically safe and effective option for alleviating chronic pain after total knee arthroplasty.

## Background

Total knee arthroplasty (TKA) is increasingly performed in the United States. Despite favorable outcomes in most cases, chronic postsurgical pain occurs in approximately 20% of patients [1]. Although residual pain has been attributed to instability, component malalignment or recurrent hemarthrosis, the underlying reason is often unclear [1]. Treatment options are limited, and most patient develop chronic pain refractory to conservative medical therapies. In recent years, musculoskeletal intervention is emerging as a minimally invasive option to address joint pain. For instance, genicular artery embolization has demonstrated early efficacy for treating osteoarthritic pain and recurrent hemarthrosis after TKA [2–5]. Similarly, genicular nerve block has been used to reduce post-arthroplasty pain, though the response is not often durable [6–9]. In this report, we describe a case of genicular artery embolization followed by genicular nerve block, with additive reduction of chronic TKA pain in a patient with recurrent hemarthrosis.

## Case presentation

This HIPAA-compliant case report was approved by the Institutional Review Board with a waiver of informed consent.

**Fig. 1:**
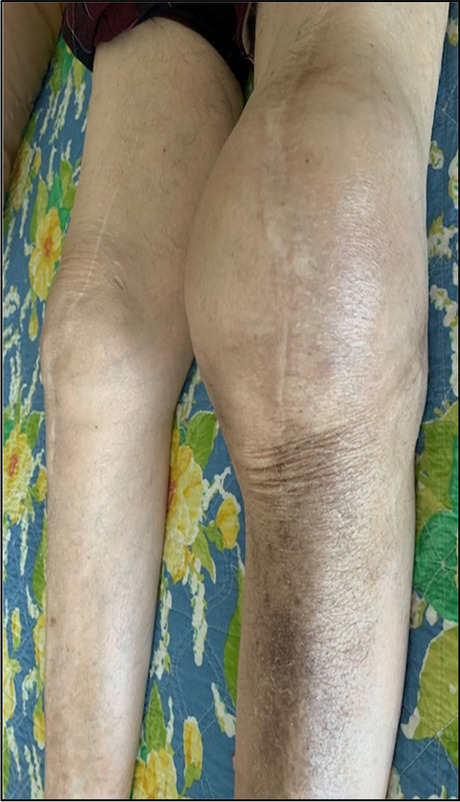
Photograph of patient knees demonstrating marked chronic swelling of the left knee relative to the right, with evidence of bilateral total knee arthroplasty

The patient was a 77-year-old man with a remote history of left knee total knee arthroplasty (TKA) complicated by infection requiring revision at age 57. For twenty years, he suffered from worsening painful left knee effusion that was refractory to conservative management, which included immobilization, application of ice packs, limb elevation, massage, and cessation of blood thinners. Both oral (e.g., non-narcotic, nonsteroidal anti-inflammatory medications, and gabapentin) and topical (e.g., lidocaine and diclofenac patches) regimens provided little pain relief. Multiple prior knee aspirations performed by the referring orthopedic surgeon demonstrated recurrent acute-on-chronic hemarthrosis with extensive intraarticular thrombus precluding complete evacuation of the joint despite aspiration of acute blood products. His laboratory workup did not show evidence of prosthetic joint infection or other infectious processes, including negative culture of the aspirate fluid, normal white blood cell counts, erythrocyte sedimentation rate (ESR), and C-reactive protein (CRP). At his initial consultation visit, the patient endorsed marked chronic swelling and severe pain (10/10 on Visual Analog Scale [VAS]) most pronounced at the left medial compartment of his knee. These symptoms were associated with limited flexion and weight-bearing, resulting in the deconditioning of his left leg musculature (Fig. 1). Preprocedural cross-sectional MR or CT imaging was not performed because of the lack of a standardized imaging protocol or metal artifact reduction available at the time of this case report.

**Fig. 2:**
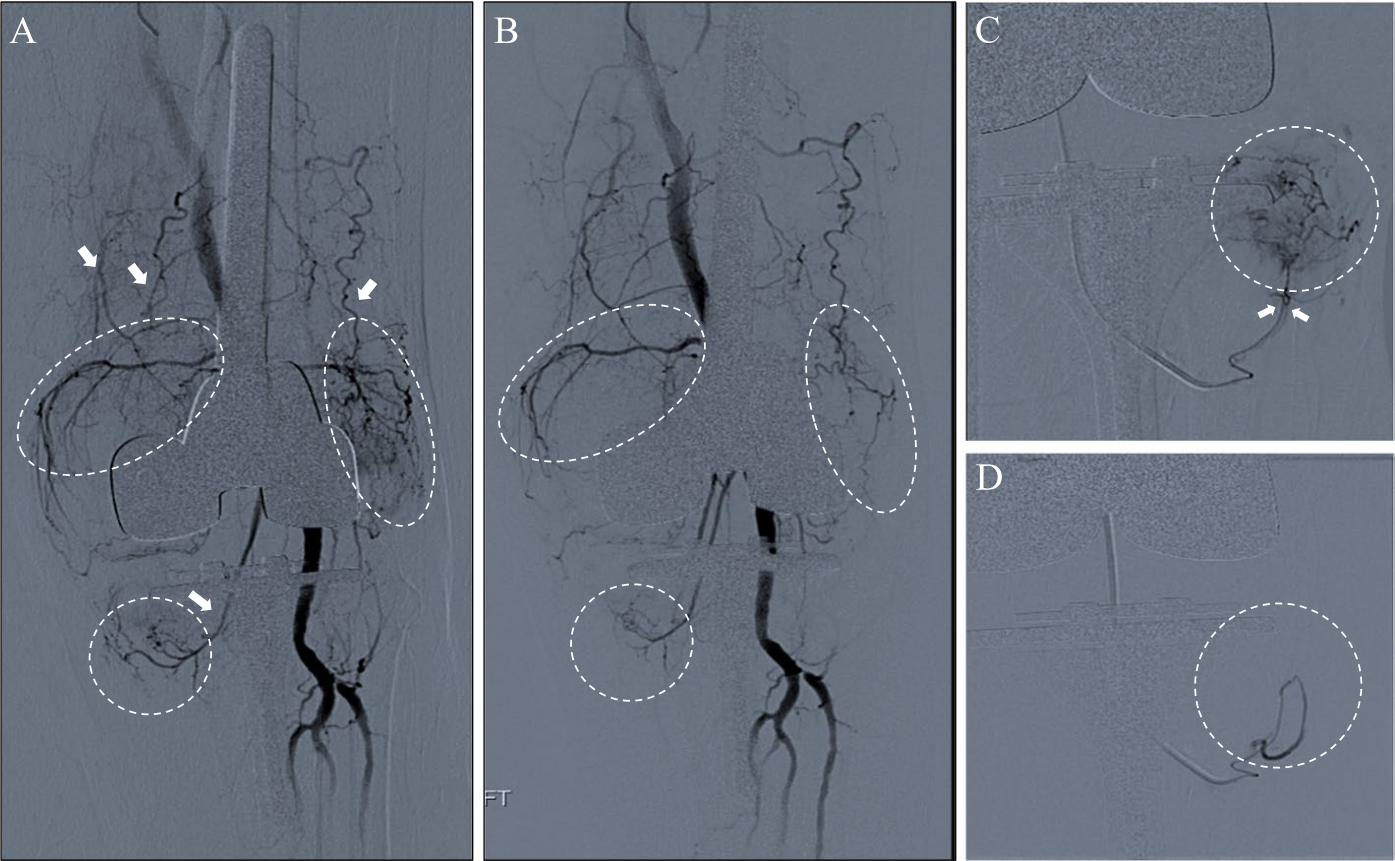
**A** Frontal projection digital subtraction angiography revealed extensive hypervascular synovium supplied by branches of the genicular arteries (white arrows) at the superomedial, inferomedial, and superolateral compartments (white circles) about the total knee replacement prosthesis. **B** Completion angiography after genicular artery embolization demonstrated subtotal devascularization with mild residual synovial blush (white circles) at the inferomedial compartment. **C** Selective angiography and embolization of the articular branches of the recurrent anterior tibial artery (white arrows) supplying the lateral joint space. **D** Completion angiography after genicular artery embolization demonstrated total devascularization

The patient underwent angiographic evaluation with the plan for genicular artery embolization to treat recurrent hemarthrosis. Under ultrasound guidance, antegrade right (contralateral) common femoral artery access was obtained, followed by placement of a 5F × 45 cm Ansel sheath (Cook Medical, Bloomington, IN) over the aortoiliac bifurcation with tip in the contralateral common femoral artery. The left lower extremity arterial system was catheterized using a 4F × 100 cm glide catheter (Terumo Medical^©^, Somerset, NJ), 2F × 150 cm Pursue microcatheter (Merit Medical Systems, South Jordan, UT), and 200 cm 0.014-inch Fathom wire (Boston Scientific^©^, Marlborough, MA). Diagnostic arteriography showed marked diffuse periarticular hyperemia about the medial and lateral joint spaces of the left knee with capsular distention (Fig. 2A). Given these findings, selective embolization was performed targeting the following genicular arterial branches: 1) the superomedial genicular artery using 0.1 vial 100–300 µm Embosphere Microspheres (Merit Medical Systems, South Jordan, UT), 2) the superolateral genicular artery using 0.1 vial of 300–500 µm Embosphere Microspheres, 3) the recurrent anterior tibial artery using 0.05 vial of 300–500 µm Embosphere Microspheres (Fig. 2B, C), and 4) an accessory superolateral genicular artery using 0.05 vial of 300–500 µm Embosphere Microspheres. Post-embolization arteriography showed marked reduction in periarticular hyperemia about the medial and lateral joint spaces (Fig. 2D). The patient had no immediate post-procedural complications.

**Fig. 3:**
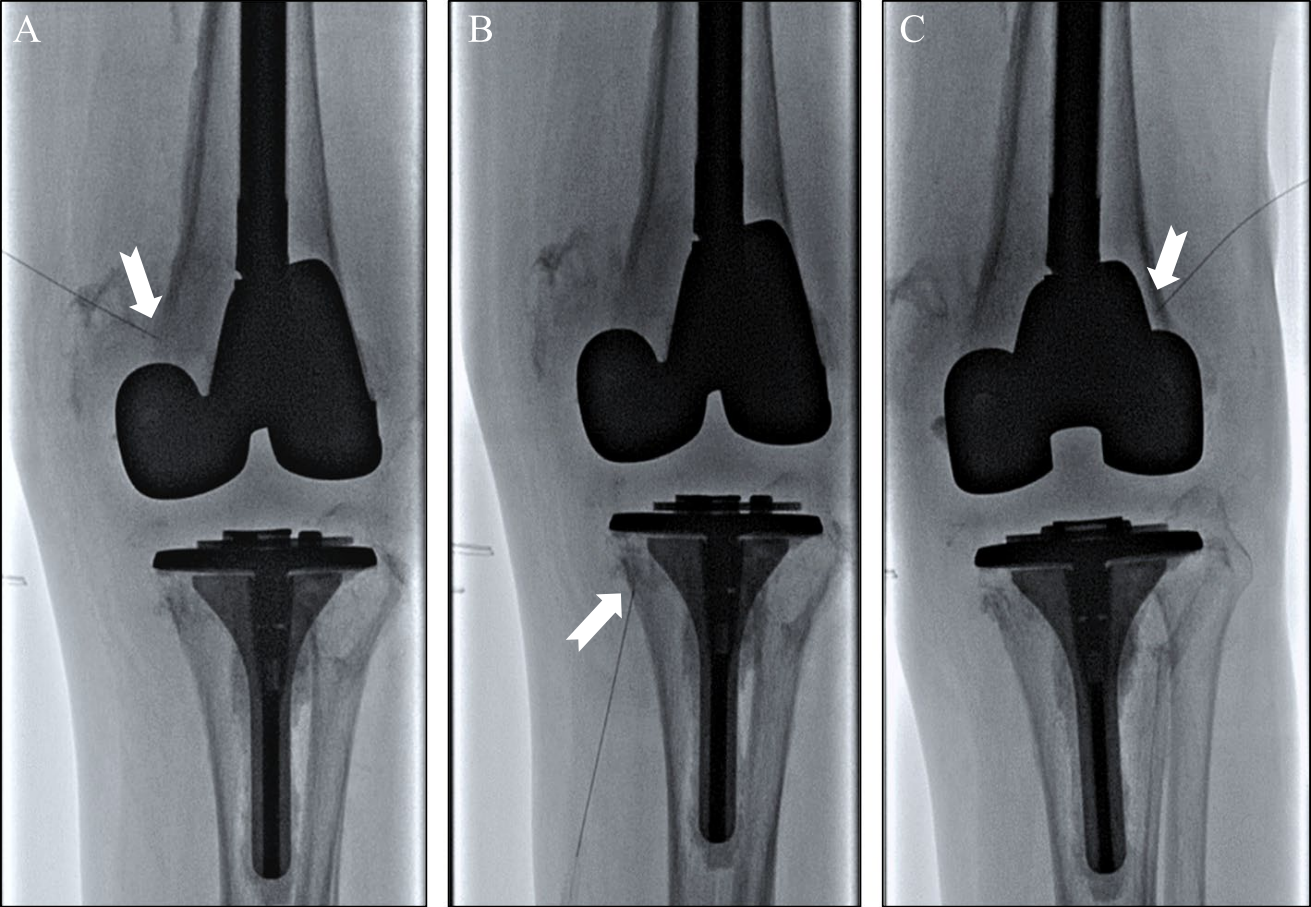
Genicular nerve block was performed in the **A** superomedial, **B** inferomedial genicular **C** and superolateral genicular nerves (dash arrows)

At the one-month follow-up, the patient reported a 50% reduction in pain (5/10 on VAS) but had persistent left knee swelling. Left knee joint aspiration removed 80 cc of sanguineous fluid, without further reduction in pain or joint distension. Culture of the aspirate fluid was negative, and the patient remains afebrile without leukocytosis. Given his persistent pain, the decision was made to proceed with genicular nerve block. Needle placement was performed under fluoroscopic guidance, with confirmed juxtacortical location in both anterior and lateral planes of the left superolateral, superomedial, and inferomedial genicular nerves (Fig. 3A–C). 5 cc of 0.25% bupivacaine was injected at each targeted location with an immediate subjective reduction in pain severity.

One month after the genicular nerve block, the patient reported reduced pain (2/10 on VAS), which was about 80% decreased compared to his initial presentation and 60% decreased compared to post-genicular artery embolization levels. This reduction in pain severity persisted through the 10-month follow-up despite limited changes in joint distention. Functionally, he improved weight-bearing and subjectively increased knee flexion. With concurrent topical lidocaine patch, the patient was able to participate in physical therapy.

## Discussion

Total knee arthroplasty (TKA) is increasingly performed in the aging population. Effective post-operative pain management plays a pivotal role in facilitating early rehabilitation and functional recovery. Chronic pain occurs in approximately 20% of patients after TKA, which is often neuropathic in nature without an explainable mechanical or infectious cause [1]. Existing conservative therapies and non-surgical interventions are largely ineffective, while revision surgery has varying success rate. As such, there is a high clinical demand for effective management options to avoid re-operation in this patient population. Musculoskeletal embolization and ablation have garnered increasing attention, supported by early safety and efficacy data for the treatment of joint pain. The treatment of post-arthroplasty pain represents another potential niche for these minimally invasive interventions, with the potential to serve an increasingly large patient population that currently has few therapeutic options.

Genicular artery embolization (GAE) is non-surgical alternative that was initially developed for treating knee hemarthrosis [2]. In recent years, this intra-arterial procedure has been adopted to selectively embolize genicular artery branches corresponding to the site of osteoarthritic pain. Conceptually, targeted endovascular occlusion reduce neovascularity and the resultant inflammation that are the important source of chronic pain. Early results from multiple studies have shown encouraging results in alleviating knee joint pain. For instance, a pilot randomized study by Bagla et al., showed that patients with moderate-to-severe knee pain refractory to conservative therapy who underwent GAE experienced significant pain reduction compared to sham treatment [3]. However, less is known about the benefit of GAE in the setting of post-arthroplasty knee pain. More recently, a case series by Chau et al., showed that GAE is safe for treating persistent pain 12 months following TKA [4]. Our case report corroborates with this result and shows a 50% pain reduction in a patient with chronic debilitating pain. Our technique differed from previous studies by using larger embolic agents (up to 500 µm), instead of the smaller embolic agents. This approach has the theoretical advantage of preventing off-target embolization caused by smaller particles reaching distal cutaneous branches that resulted in skin discoloration or ulceration.

Genicular nerve block (GNB) is routinely used to alleviate pain after TKA in the post-operative setting and for osteoarthritic knee pain [6–8]. This percutaneous intervention can be performed using anatomical landmarks and fluoroscopic guidance to target the sensory innervating branches of the knee, including the superomedial, inferomedial, superolateral, inferolateral genicular nerves. Neurolysis can be achieved with ablation (e.g., radiofrequency) or analgesic/corticosteroid injection, without affecting the motor function. GNB was first used for chronic osteoarthritic knee pain [6], then later adopted for post-operative TKA pain due to significant pain reduction and functional improvement in these patients. A randomized trial by Rambhia et al., showed that GNB was associated with a significant reduction in opioid consumption in patients after TKA [7]. Qudsi-Sinclair et al., showed both ablation and analgesic/corticosteroid injection are equally effective for pain reduction and joint function improvement the first 3 to 6 months [8]. Building upon these experiences, our case report further expands the potential benefit of GNB in patient with chronic post-TKA pain. Our case report is unique in the sequential application of both modalities, resulting in additive pain reduction in a patient with longstanding post-total knee arthroplasty (TKA) pain and disability (20 years). The sustained pain reduction for at least 10 months is noteworthy, considering the persistence of objective joint distention and continued hemarthrosis.

Post-TKA pain is multifactorial, mediated not only by hemarthrosis but also encompassing processes such as hypertrophic synovitis and neuropathic pain with peripheral and central sensitization [9]. The successful outcome of this case report highlights a potential additive effect in symptomatic pain relief when both devascularization and regional neural blockade are sequentially employed. Additionally, this case report supports a new role for embolization in the treatment of post-TKA pain, through a mechanism that may be similar to that used to treat osteoarthritis (OA) [10]. From a mechanistic standpoint, the persistent hemarthrosis following GAE raises the possibility that pain symptoms are not solely mediated by blood products within the knee joint. Instead, it is plausible that inflammation from the resultant hypertrophic synovitis is another major source of pain symptoms, similar to OA. As such, the degree of hyperemia observed on pre-procedural CTA or MRA could help tailor embolization and nerve block treatments to address local inflammation. Additionally, our finding of a combination approach for the management of post-TKA pain should be explored further, given the complex etiologic processes (e.g., synovial inflammation, angiogenesis, neurogenesis, and peripheral and central sensitization, among others). Our findings incentivize prospective studies to validate the effectiveness and durability of the combined GAE and GNB to alleviate chronic, debilitating pain following total knee arthroplasty.

## Conclusion

We advocate that the combination of genicular artery embolization and genicular nerve block may be a technically safe and effective option for alleviating chronic pain after total knee arthroplasty.

## Data Availability

Not applicable.
